# Financing the Agri-Environmental Policy: Consequences on the Economic Growth and Environmental Quality in Romania

**DOI:** 10.3390/ijerph192113908

**Published:** 2022-10-26

**Authors:** Nicoleta Mihaela Doran, Roxana Maria Bădîrcea, Marius Dalian Doran

**Affiliations:** 1 Faculty of Economics and Business Administration, University of Craiova, 13 A.I. Cuza, 200585 Craiova, Romania; 2 Doctoral School of Economics and Business Administration, West University of Timisoara, 300223 Timisoara, Romania

**Keywords:** CO_2_ emissions, irrigation arrangements, drainage arrangements, chemical fertilizers used in agriculture, improving and combating soil erosion, natural fertilizers used in agriculture, pesticides used in agriculture

## Abstract

The aim of this research is to point out the impact that the application of the agri-environmental policy has on the economic growth and on the quality of the environment, these being the main aspects targeted by the practice of a sustainable agriculture. The research is conducted based on the agri-environment indicators for Romania for the period of time between 1997 and 2019. In order to answer the objectives of this whole research, we performed stationarity tests, a cointegration test and used the Fully Modified Least Squares (FMOLS) method to estimate the relationships between the variables included in the three proposed models. The obtained results highlighted the positive influence exerted by the area that was arranged for irrigation and the agricultural area that was arranged with drainage works on the GDP, but also the negative influence of the amount of natural fertilizers used in agriculture. The use of chemical fertilizers and pesticides generates an increase in environmental degradation, meaning CO_2_ emissions, while an increase in the agricultural area arranged with erosion control and land improvement works, leads to reducing environmental degradations. The limitations of this research lie in the fact that the agri-environmental indicators are specific to each country in the European Union and, therefore, it is difficult to make comparisons with other member states or to apply the measures recommended for Romania to other states with similar agricultural and economic systems.

## 1. Introduction

According to the statistics [1], agriculture represents less than 2% of the gross domestic product (GDP) and less than 6% of employment at the Organization for Economic Co-operation and Development (OECD) level, however, in many developing countries, it represents 30% of the GDP and two thirds of employment. In order to ensure food supplies for the entire world population that is estimated for the year 2050, we need agricultural production to record a raise of 70%, which would mean an agri-environmental policy that leads to increasing productivity and efficiency in this field, which at the same time ensures protection of the environment. In the next period, satisfying the request for agricultural products will be much more difficult than before, on the one hand due to global climate changes that destabilize many natural processes, but which are necessary for modern agriculture and on the other hand, due to the fact that the techniques that farmers rely on to increase production cause damage to the environment. At the same time, at a European level, the European Green Deal aims to have Europe neutral from a climatic point of view by 2050, and in order for that to become possible, the European Climate Law was adopted, which has a more ambitious objective, namely, the net reduction of greenhouse gas emissions by at least 55% by 2030, compared with the levels from 1990 [2].

The implementation of the European Green Deal and applying the strategies on the agri-food chain and the environment is undertaken through agri-environment and climate measures. The intensification and modernization of agricultural practices in recent decades have put great pressure on the environment and, in particular, on biological diversity. Starting in 1992, several initiatives and measures have emerged in the Common Agricultural Policy (CAP) aimed at reducing these pressures, alongside agri-environmental measures. They are not standardized at the European level but are developed on a national level by each EU Member State. Agri-environment packages include measures such as: a reduction in fertilizers and pesticides, the establishment of animal loads on meadows, and the protection and improvement of habitats necessary for wildlife species.

Although agri-environmental measures have been introduced to reduce the negative effects of agriculture on European habitats and wildlife (but also to contribute to the EU’s goal of halting biodiversity loss), the results of several studies have shown that that they have not lived up to expectations and have, so far, brought too few results compared to the volume of financial resources invested [3,4,5,6].

In Romania, the share of agriculture in the GDP in 2021 was 4% (decreasing as compared to previous years) but with a high potential for growth and investment, and the share of the population employed in this sector was 20.5% [7], compared to an average of 4% in the EU. Romania’s agricultural area covers 14.6 million hectares of land, of which only 10 million are occupied by arable land. In the last 30 years in Romania, the focus of development in agriculture was on the expansion of operational equipment and the construction of necessary buildings, while topics such as environmental protection, erosion prevention, measures against the effects of climate change and species protection played only a subordinate role.

The challenge currently consists in preserving this rich Romanian resource that Romania owns compared with other EU state members—the biodiversity associated to agricultural fields that are facing the socio-economic changes that are expected in rural areas in the future. In order to ensure that these objectives are met, extensive agricultural systems (based on reducing inputs and on the sustainable use of natural resources) need to be supported in order to be able to compete both with the pressure to adopt intensive agricultural systems and also in a wider context, with other emerging economic activities with which agriculture will be able to compete from the perspective of agricultural land use. At the same time, the continued encouragement of agricultural activities in areas with a high environmental value, but especially in areas facing natural constraints and in mountain areas, as well as the avoidance of an abandonment of these areas, must be a priority in the future. The environmental and climate measures of the National Rural Development Program (NRDP) 2014–2020, add up to 30% of the total European Agricultural Fund for Rural Development (EAFRD) allocations, consisting of both permanent natural and semi-natural grasslands and extensively used traditional orchards or arable land. This has promoted the practice of agriculture that involves an avoidance or limitation of the use of heavy machinery and an avoidance of chemicalization, together with the enforcement of traditional agricultural techniques used (which have basically been reduced to non-intensive grazing and the establishment of some data and methods of mowing), while favoring the maintenance of priority habitats and important species with a traditional cultural background, as well as a rational use of natural resources.

The objective of the present research is to point out the impact the application of the agri-environmental policy has on the economic growth and of the quality of the environment, these being the main aspects targeted by the practice of a sustainable agriculture. 

## 2. Literature Review

### 2.1. The Effects of the Agri-Environmental Policy on the Economic Growth

A solution to solving the problem of the growing necessity for agricultural products which is directly corelated to population growth and to economic growth, was identified by Ruttan [8] as being a technological change, which has its roots in economic growth and in state development. Technological advances in agriculture will, thus, lead to increasing productivity but must ensure protection for the environment at the same time [1]. Following that, economic growth that ensures environmental safety, thus, depends both on public investments and on private investments in the agricultural sector. Other identified effects of agriculture are those of increasing the well-being of rural populations and reducing poverty, especially in the less developed countries, increasing foreign exchange and stimulating international trade. Moreover, agriculture plays a major role in the use and management of natural resources. Blandford noted the great importance that agriculture has both economically and ecologically, in terms of the value of environmental services to which it contributes [9].

Buckwell, Heissenhuber and Blum [10] concluded that sustainable intensification in agriculture is the solution to combining an intensive and productive agriculture industry, while still respecting the required environmental standards. This intensification will lead to the achievement of the objectives proposed by the European Commission and to ensuring internal and external competitiveness.

In a paper analyzing agri-environmental policy in Poland, Jezierska-Thöle, Gwiaździńska-Goraj and Dudzińska [11] reached a result that demonstrated a close correlation between payments through agri-environmental policy and the economic dimension of the quality of life, thus, demonstrating the important role played by agriculture in ensuring a decent living. Gollin, Hansen and Wingender [12] studied the economic effects of increasing agricultural productivity in the developing world and identified unequally distributed positive effects of agricultural productivity on the GDP, food yields, the level of education and life expectancy in different countries. A study [13] conducted in Romania analyzed the relationship between Common Agricultural Policy, rural development and the general process of growth and economic development in the rural areas of Romania, and it was pointed out that there was a positive influence from the funding for agriculture on the economic growth in rural areas.

### 2.2. The Effects the Agri-Environmental Policy on the Quality of the Environment

The accession of papers analyzing the effects of agriculture on the environment [9,14] showed that views are contradictory, because the effects can be both positive and negative. For example, Zhang et al. [15], Hezri and Ghazali [16], and Fridman and Kissinger [17] demonstrated that intensive agriculture has led to massive deforestation, high levels of greenhouse gas emissions and soil depletion. Similarly, OECD statistics show that the agricultural sector, together with forestry and other land uses, accounts for almost a quarter of all anthropogenic greenhouse gas (GHG) emissions.

In another work, Tubiello et al. [18] pointed out the fact that over a period of 50 years, the greenhouse gas emissions from agriculture and forestry have doubled, which raises questions about the evolution towards 2050, when the population growth will require more and more food products.

To provide the food needed worldwide and to maximize crop yields, large amounts of chemical fertilizers have been used. This has led to severe soil degradation, soil compaction, a reduction in soil organic matter and a loss of soil carbon, along with reduced yields of chemical fertilizers on crops [19].

In Romania, the consumption of mineral fertilizers represents 5% of the total EU consumption; therefore, compared to the EU average of 77.2 kg of nitrogen fertilizer consumed per hectare in 2018, Romania applied less than 60 kg [20,21].

The total elimination of the use of mineral fertilizers is not possible because this would lead to a drastic decrease in food production. That is why the widespread use of natural fertilizers that ensure high yields and minimize the effect on the environment is required. In Romania, it was found that more than 90% of the amount of fertilizer applied was represented by natural fertilizers, explained by the fact that they are easily available and are inexpensive, and most often come from the existing animal husbandry activities. Another explanation would be that chemical fertilizers have a higher price and farmers are not willing to allocate large funds for them [22].

A study [23] on soil drainage claims that adequate drainage facilitates plowing and early planting, can lead to longer crop growth periods, provides more water and nutrients from the soil, can decrease soil erosion, and can lead to higher and richer yields.

An analysis [24] of the implications of drainage infrastructure for sustainable development shows that it enables efficient water management by reducing the flow and, thus, the nitrates in agricultural areas and that it helps to mitigate the effects of climate change on agriculture by reducing the risk of drought and floods.

The negative implications of agriculture have also been identified in the fields of maintaining biodiversity, soil erosion, providing necessary food products and improving climate change [25,26,27,28].

Soil erosion results in the loss of fertile soil, a reduction in agricultural productivity and, therefore, the supply of food necessary for a growing population [29].

The monitoring of soil quality in Romania revealed a series of problems regarding the use of land in Romania, through the manifestation of a moisture deficit, salinization and alkalinization, through soil erosion, a reduction in the content of organic materials, soil acidity, compaction, pollution, etc. [30].

An important problem for the environment is also the use of pesticides, since they can cause contamination of the environment and food. At the same time, however, pesticides play an essential role in reducing diseases and increasing harvests worldwide. The studies carried out to analyze the evolution of pesticide use have highlighted the fact that Romania is in last place at the European level, with the lowest consumption of pesticides per ha, and the conclusion is that pesticides must be used while taking into account local conditions, at the regional level and at the farm level [31].

A series of environmental problems determined by the development of irrigated agriculture were highlighted such as: human resettlement, the loss of biodiversity, watershed degradation, soil erosion and fertility [32]. Estimates have been made of the area of land that will need to be irrigated to provide food for a population of more than nine billion people in 2050, and it was concluded that it will generate an expansion of agriculture in natural ecosystems in search of water [33]. Another analysis carried out to assess the environmental impact of irrigation works alongside the production benefits concluded that the use of irrigation can increase crop yields and reduce water consumption, but leads to increased carbon emissions [34].

In Romania, after abandoning the centralized irrigation systems, agriculture has remained dependent on the weather. Climate changes faced by Romanian farmers, such as drought or floods, pose a risk to the water resources and mitigation actions are considered necessary [35]. According to the statistics [36], Romania has an area designed for irrigation of approximately 3.1 million hectares, although the irrigated area differs from year to year depending on the rainfall and existing facilities.

In a study that analyzes the factors that influence agricultural production in Romania [37], it was observed that one of the influencing factors was the level of irrigation norms (i.e., irrigation norm-translated) whose growth in Romania requires institutional, political and investment involvement.

Based on these shortcomings, the agri-environmental policy was designed to contribute to mitigating the effects of agriculture on the environment. Analyses of the effectiveness of agri-environment policies have also led to conflicting results [38]. For example, on one hand, subsidies can lead farmers to adopt modern means to help them in the production process and increase productivity, while on the other hand, they can also be a demotivating factor because they allow activities to be carried out at the limit of inefficiency. Positive effects of agriculture on the environment have also been highlighted, such that Stevens [1] considers that it is possible for agriculture to provide a number of environmental and ecosystem services that can positively influence the environment. We can say that the main positive effect is determined by the ability of agriculture to reduce greenhouse gases by trapping carbon in the soil and vegetation with the potential to eliminate up to 20% of the global fossil fuel emissions; however, all this depends on the ability to manage the use and cultivation of the land, in order not to reach higher emissions caused by an in-appropriate use of agricultural land. In conclusion, in this sense of the positive effects, Nor Diana et al. [39] believes that agriculture can contribute to green growth; thus, ensuring both economic growth and sustainability, but also preventing the destruction of the ecosystem, a loss of biodiversity and the wastage of natural resources.

To ensure a positive influence on the agri-environmental policies, the need to stimulate the production of environmental goods, or “ecosystem services” has been identified by Kirchner et al. [40], Merckx and Pereira [41], and Bethwell et al. [42], meaning carbon storage, increasing the resistance to natural disasters, pollination and soil functionality [43], as well as ensuring habitat preservation and the control of invasive species.

Batáry et al. [44] examined the agri-environmental schemes before and after the 2007 review and came to the conclusion that the revised schemes have not been more effective than the schemes that were implemented before the review. The agri-environmental schemes can be efficient for wildlife conservation on agricultural land, but they are expensive and need to be addressed very carefully. In Slovenia, increased production, especially in the beef and dairy sectors, which was supported by direct payments of the common agricultural policy, and a forest succession in marginal areas have been identified as potential key factors in the recent loss of agricultural land biodiversity in Slovenia [45]. In another study, Biffi et al. [46] found that higher spending on agri-environment schemes was associated with areas with low levels of soil organic carbon and high greenhouse gas emissions in both the US and the EU, with inconsistencies identified between the funding and environmental needs, namely, not targeting those areas with the highest water stress, that were threatened with biodiversity loss, soil erosion or nutrient runoff.

Based on the analyzed papers, we observed that there was interest in the effects that the agri-environmental policy has generated on economic growth and on the environment at the European level or in various countries. In the literature dealing with agri-environmental issues at the Romanian level, only indicators related to the agri-environmental policy were analyzed separately, and not necessarily in relation to the effects generated on the economic growth, but especially on the environment. Thus, this paper covers this gap identified in the literature because it analyzes the effects generated by the agri-environmental policy applied in Romania on economic growth and on the quality of the environment. 

To achieve the main purpose of this study and cover the gap in the field at a national level, two research hypotheses were formulated:

**H1.**  *A* *gri-environmental policy impacts the economic growth through the means of soil care and protection.*

**H2.**  *The application of the agri-environmental policy generates effects on environmental degradation and sustainability.*

## 3. Description of the Variables and Data Series

According to the proposed objective and to confirm the hypotheses formulated, we used data from 1997 to 2019, collected from the Eurostat database and the database of the National Institute of Statistics in Romania. In the research, we used the GDP growth (GDP) as the dependent variables to assess the economic growth, while the environmental quality was assessed through two dependent variables, which were: environmental degradation (ED) and environmental sustainability (ES), as proposed by Bhutta [47], whose description can be found in Table 1.

At the level of Romania, the system of indicators related to the agri-environmental policy includes seven categories of indicators, which represent the independent variables included in the study and they are described in Table 2. These indicators were selected from the database of the National Institute of Statistics in Romania depending on the availability of data for the time period under analysis. The set of indicators on the agri-environmental policy is established by each EU Member State independently of the rest of the Member States, based on the recommendations made by the European Commission.

## 4. Functional Form of Variables and Econometric Techniques

In order to meet the research objective, we combined the research model which surmises the environment in terms of quality and sustainability proposed by Bhutta [47], with the research models which include variables that characterize the agri-environmental policy proposed by other authors [48,49,50,51,52]. The results of these combinations led to the development of three econometric models that can respond to the formulated hypotheses and cover the gaps in the literature in the field:

Model 1: GDP = f(AE1, AE2, AE3, AE4, AE5, AE6, AE7)

Model 2: ED = f(AE1, AE2, AE3, AE4, AE5, AE6, AE7)

Model 3: ES = f(AE1, AE2, AE3, AE4, AE5, AE6, AE7)

The mathematical equation of the multiple linear regression which is needed to identify the influence of the agri-environmental indicators on the economic growth and environmental quality related to the proposed models is of the form:

*Y_t_* = *α* +*β*_1_*AE1_t_* + *β*_2_*AE2_t_* + *β*_3_*AE3_t_* + *β*_4_*AE4_t_* + *β*_5_*AE5_t_* + *β*_6_*AE6_t_* + *β*_7_*AE7_t_* + *ε* (1)

where *Y* is a dependent variable and can be the GDP, ED or ES, α represents the free term of the equation, *AE1*…*7* represents the seven indicators of the agri-environmental policy in Romania, *β*_1…7_ represents the coefficients associated with the independent variables and ε represents the standard error.

The appropriate methodology for the present research involved performing the stationarity tests (ADF and PP), the cointegration test (Engle–Granger) and then estimating the relationships between the variables included in the three models using the Fully Modified Least Squares (FMOLS) method. Engle and Granger [53] pointed out that a linear combination of two or more non-stationary series may be stationary. If such a stationary linear combination exists, the non-stationary time series are said to be cointegrated. The stationary linear combination is called the cointegrating equation and may be interpreted as a long-run equilibrium relationship among the variables. Phillips and Hansen [54] proposed an estimator which employs a semi-parametric correction to eliminate the problems caused by the long run correlation between the cointegrating equation and stochastic regressors innovations. The resulting Fully Modified Least Squares (FMOLS) estimator is asymptotically unbiased and has fully efficient mixture normal asymptotics allowing for standard Wald tests using an asymptotic Chi-square statistical inference.

## 5. Results and Discussion

The summary statistics in Table 3 show that the average of the GDP annual growth in Romania was 3.2% for the period under consideration. The mean statistic in the table further reveals the average carbon emission to be 86,540 kt, while the average adjusted net savings (excluding the particulate emission damage) had a negative value of −0.08% of GNI. With respect to the statistical distribution of the series, the economic growth (GDP), environmental sustainability (ES), AE4 and AE6 all appeared to be negatively skewed, while the result were otherwise (i.e., positively skewed) for environmental degradation (ED), AE1, AE2, AE3, AE5 and AE7. For the kurtosis statistic, we found it to be mostly platykurtic for all the series except for AE4, AE5 and AE7. Finally, we can see that the computed probability values attached to the Jarque–Bera normality test statistic were larger than the various conventional chosen levels of significance (1%, 5% and 10%) for seven variables, which by implication suggests a non-rejection of the non-normality across the individual series under consideration.

According to the standard procedure, particularly when modelling with a time series, each of the variables must be subjected to, at most, one stationarity test. Essentially, both the Augmented Dickey–Fuller (ADF) and Phillipes–Perron (PP) test were explored. The unit root tests performed on the earlier defined measures for each of the variables and the outcomes are represented in Table 4.

The results obtained both by applying the ADF unit root test and by applying the PP test (Table 4) show us that all the variables were stationary in the level (GDP, AE4 and AE7) or at the first difference (ED, ES, AE1, AE2, AE3, AE5, AE6). After examining the stationary properties of the data, the study employed a correlation matrix to confirm that the data for the current investigation were free of the problem of collinearity. Table 5 presents the results of the correlation coefficients, which show that there was no problem of multicollinearity in the data because the coefficient of correlation among any two variables was less than 1.00.

The Engle–Granger tau-statistic (Table 6) and normalized autocorrelation coefficient (which we term the z-statistic), both reject the null hypothesis of no cointegration (unit root in the residuals) at the 5% level for all the three models. On balance, the evidence clearly suggests that the variables in the model were cointegrated.

**Figure 1:**
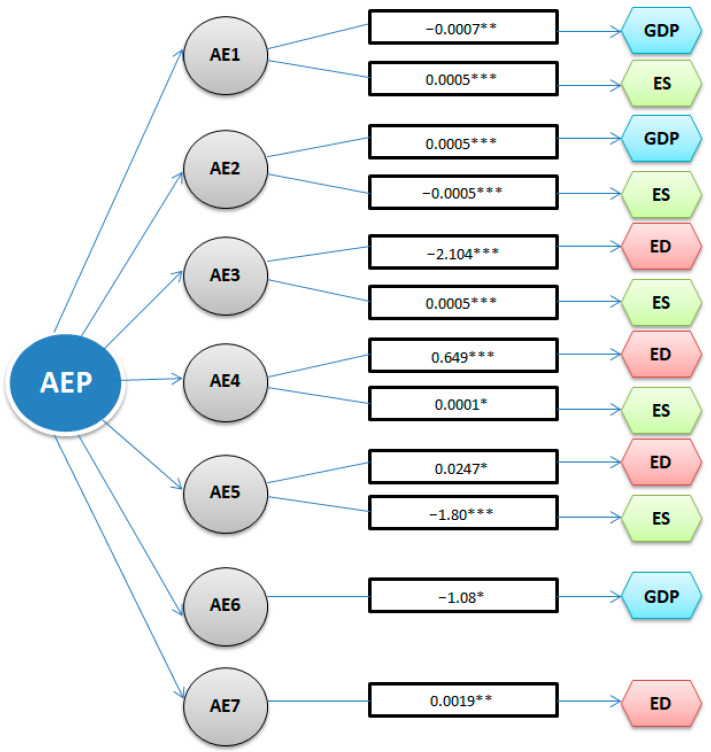
The influences of the agri-environmental indicators on economic growth and environmental quality in Romania. Note: *, **, *** represents the significance level of 1, 5 or 10%.

Figure 1 showed the influences, statistically significant, determined by the evolution of agri-environmental indicators on the economic growth and environmental quality in Romania.

In the case of the first proposed econometric model, we can observe the influence of three variables over the GDP, which are: the arranged area for irrigation (AE1), the agricultural area arranged with drainage works (AE2) and the quantity of natural fertilizers used in agriculture (AE6).

Romania presents a higher risk to climate change, the effects of which being clearly reflected in the changes of temperature and rainfall. The droughts, the floods, and other issues related to climate change have a significant impact on the stability of production and on national food security and a lack of adequate infrastructure contributes to limiting economic development despite the potential of agriculture.

The old irrigation arrangements generate a high consumption of water and energy, which has a negative impact on the water reserves of Romania, a country included in the category of countries with low water reserves. Its irrigation facilities are in an advanced stage of degradation and on 75% of the surface of these facilities, irrigations are not functional while the functional ones are inefficient in terms of their water and energy consumption, as well as being expensive for farmers. It is estimated that about 11% of the country’s agricultural area is covered by economically or marginally viable irrigation networks.

Unfortunately, the evolution of the indicator AE1 shows us the lack of interest for improving the irrigation systems in Romania, whereas during the analyzed time interval, a continuous decrease in the agricultural areas provided with irrigation systems was observed, except for the year 2020, the only year in which there was an increase in the indicator compared to the previous year. All this explains the negative influence that the AE1 indicator has on the evolution of the GDP. These results are in agreement with previous research that considers irrigation to be a factor that can contribute to an increase in agricultural production, and implicitly the contribution of agriculture to the GDP [37,55].

The establishment of drainage systems (AE2) and their use has as its main purposes: minimizing or preventing the impact of climate change on floods; the avoidance of damages caused by the flooding of agricultural lands; increasing the quality of life by reducing the damage caused by floods; maintaining complex economic activities in areas protected from floods; and reducing potential economic losses caused by floods. Unfortunately, the drainage systems in Romania are mostly damaged, with the AE2 indicator registering lower and lower values over the analyzed time interval (the reason for why its influence on the economic growth is very low), and this is why the stakeholders involved in this field must give greater importance to drainage works to increase the quality of the soil alongside irrigation, combating erosion and desiccation.

A significant negative influence is given by the AE6 indicator on the evolution of the GDP. The use of natural fertilizers is associated with an ecological agriculture, i.e., an agricultural system which avoids the use of artificial fertilizers, pesticides or herbicides and which uses organic fertilizers and organic methods of crop rotation. Natural fertilizer minimizes the negative impact on the environment and is cheaper than artificial fertilizer; however, due to the fact that they do not have a quick and visible contribution to agricultural production, this category of fertilizers is very rarely used by large agricultural producers, who expect to obtain aesthetically pleasing products in a quick but unhealthy way, that will bring high incomes in the short term. They are widely used by small producers, whose production, however, does not have a large contribution to the GDP [22]. At the same time, the practice of high prices for ecological products compared to other products on the market reduces the demand for them.

Therefore, the influences presented in Figure 1 confirm the first hypothesis H1, regarding the impact of the agri-environmental policy on economic growth through three indicators: AE1 and AE6 that negatively influence the economic growth and AE2 that releases a positive impact on economic growth.

At the same time, the results show that the use of chemical fertilizers and pesticides (AE5 and AE7) generate an increase in environmental degradation, such as CO_2_ emissions; however, compared to other EU countries, in Romania, the use of chemical fertilizers is quite low, but given the fact that we want to increase the productivity of the agricultural sector, it is important that this increase does not lead to a wider use of these fertilizers. These results justify the actions taken by the European Commission [56] to bring about a 20% reduction of fertilizer use by 2030.

The environmental sustainability (ES) is positively influenced by three agri-environmental indicators (AE1, AE3 and AE4) and negatively by two agri-environmental indicators (AE2 and AE5). Major negative changes can be seen in the case of AE5 indicator-chemical fertilizers used in agriculture, which at an increase of one unit will determine a reduction by 1.8 units of environmental sustainability. The EU laws that refer to chemical products ensure the primary protection for the health of both humans and the environment. This ensures stability and predictability for companies operating in the internal market. In 2016, the European Chemicals Agency [57] (ECHA) published a report on the functioning of the REACH Regulation and the CLP Regulation, which indicated that law enforcement activities were constantly evolving.

Therefore, regarding the second hypothesis H2, we can see that, indeed, this is confirmed by the fact that five of the analyzed agri-environmental indicators (i.e., AE1, AE2, AE3, AE4 and AE5) influence environmental sustainability (ES) and four of the agri-environmental policy indicators (i.e., AE3, AE4, AE5 and AE7) influence environmental degradation (ED).

Although the results obtained support the idea promoted by the EU’s policy of reducing the use of chemical fertilizers to ensure the protection of the environment and biodiversity, we still agree with those who believe that in order to ensure the sustainable development of agriculture, the reduction in the use of chemical fertilizers must be adapted to the actual average consumption, which in Romania is quite small [20,21], and it must also take into account the local needs of each country in terms of increasing productivity and farmers’ incomes.

## 6. Conclusions

In the context of a dynamic world and events strongly marked by global climate change and the danger of environmental degradation, there is a clear goal, namely, to guide sustainable development on a fair path and to protect environmental factors, thus including vigorous efforts towards mandatory measures in response to environmental challenges due to climate change. In this sense, the objective of this article was to determine the influence exerted by the application of agri-environmental policy on the economic growth and environmental quality in Romania.

As much as the level of public awareness of the environmental footprint of all sectors of the economy and agriculture, has increased, we cannot help but notice a growing challenge for governments to assess and address these influences. If the Romanian Government succeeds in conceiving agricultural policy measures capable of ensuring a synergy between increasing the productivity of the agricultural sector and environmental performance, no further measures will be needed to counteract the negative effects on the environment, which can ensure a lower cost in the fight against environmental degradation. 

Agriculture has become one of the most vulnerable sectors to climate change, and the estimates for the future predict that these tendencies will intensify. The current irrigation system continues to face several issues, with the current placement and the technical conditions of the irrigation infrastructure leading to a higher price for water, which is affordable mostly to only large, commercial farmers, but which is prohibitive to small farmers. In addition to climate changes, Romania is facing a few other environmental issues, highlighted by the deterioration of its soil and water quality in the last decades. The production for renewable energy that comes from agriculture and the areas of land cultivated in organic farming are increasing and these are areas that need to be further developed in the future.

This study provides several clues on how to further improve the design and the adoption of agri-environmental measures, which should allow and ensure a higher effectiveness and protection for the environment in the future. These findings may help to improve the EAFRD allocation mechanisms and may identify opportunities to improve the future targeting of ESA expenses. The European rules regarding European Structural and Investment Funds oblige Member States to promote the environment and climate in the framework of their financing strategies and programs for economic, social and territorial cohesion, rural development and maritime politics. 

Achieving sustainability involves mobilizing public and private sources of funding. The use of European Structural and Investment Funds (e.g., the European Regional Development Fund (ERDF), Cohesion Fund (CF), European Social Fund (ESF), European Agricultural Fund for Rural Development (EAFRD) and European Fisheries and Maritime Fund (EMFF)) is essential for countries to achieve their environmental goals and for their integration into other policy areas. Other instruments, such as Horizon 2020, LIFE and the European Fund for Strategic Investments (EFSI), can support the implementation and dissemination of good practice.

Thus, we have identified the need to make investments in the rehabilitation of the main irrigation infrastructure in order to generate macroeconomic effects that mostly consist in gaining an economic growth at the level of the agricultural sector as compared to the situation before the rehabilitation as a result of:

- Improving the productivity of land, currently deficient in moisture, saline, acids, etc.
- Improving the structure of the crop plan, through using valuable and profitable plants, and reducing the use of pesticides and fertilizers.
- Increasing the use of natural fertilizers and raising public awareness of the benefits of consuming organic products.
- Increasing the average production per hectare through irrigation.
- Simultaneously with investments for irrigation, larger investments in drainage works will be necessary which, as we have shown, have not been used on a large scale in Romania and which can lead to an increase in productivity and farmers’ incomes.
- Although all the official documents identified the need for soil erosion mitigation works in Romania, which is considered to be the most serious hazard with medium- and long-term consequences, we believe that greater efforts and investments are needed in this regard. We have also identified the need for large farmers to use natural fertilizers as well, which will likely lead to positive effects on the GDP and generate visible positive effects on environmental sustainability. At the same time, the use of pesticides will have to be undertaken very carefully and adapted to each individual farm [31].

Under the national rural development program, EAFRD funds amount to EUR 3.522 billion—with 40% of the total budget allocated to environmental protection measures, but only 11% to agri-environmental measures. 

Romania will be concentrating on limiting its GHG emissions and on managing environmental threats to natural resources in agriculture, by promoting modern production technologies, practices and products which do not harm the environment. Investments in green technology will be encouraged. Likewise, particular attention will also be paid to the promotion of organic products, support for renewable energy sources and afforestation of low-quality and unproductive agricultural land. In solving the environmental issues, Romania will need to take into account the opportunities offered by the CAP, while fulfilling at the same time its European and international commitments.

## Figures and Tables

**Table 1:** Dependent variables description.

Variable	Indicator	Unit of Measure	Details
Economic Growth (GDP)	Annual GDP growth	percentage	Annual percentage growth rate of GDP at market prices based on constant local currency. Aggregates are based on constant 2015 prices, expressed in USD. GDP is the sum of the gross value added by all resident producers in the economy plus any product taxes and minus any subsidies not included in the value of the products. It is calculated without making deductions for the depreciation of fabricated assets or for the depletion and degradation of natural resources.
Environmental degradation (ED)	CO_2_ emissions	kilotonnes	Carbon dioxide emissions are those stemming from the burning of fossil fuels and the manufacture of cement. They include carbon dioxide produced during the consumption of solid, liquid, and gas fuels and gas flaring.
Environmental sustainability (ES)	Adjusted net savings	% of GNI	Adjusted net savings are equal to net national savings plus the education expenditure and minus the energy depletion, mineral depletion, net forest depletion, and carbon dioxide. This series excludes particulate emissions damage.

**Table 2:** Independent variables description.

Variable	Indicator	Unit of Measure	Details
AE1 Irrigation arrangement	The agricultural area arranged with irrigation	hectares	Represents the ensemble of works carried out in order to ensure the controlled supply of water, of the agricultural crops in order to increase the agricultural production and to ensure its independence from the meteorological conditions.
AE2 Drainage arrangements	The agricultural area arranged with drainage works	hectares	Represents the totality of the hydrotechnical works carried out for the cut of the excess water from the surface of the lowlands in order to cultivate them or for sanitary prophylactic reasons.
AE3 Improving and combating soil erosion	Agricultural area arranged with works of combating erosion and land improvement	hectares	Represents the complex of hydrotechnical works performed to reduce or to stop the degradation of the soil surface by removing its fertile layer under the action of external geographical agents, and carrying out regularization works to avoid rainwater runoff from the slopes to avoid damage caused by floods on the land of the slope.
AE4 Drainage arrangement	The agricultural area arranged with drainage works	hectares	Represents the totality of hydrotechnical works for the removal of excess moisture and consolidation of a land on an agricultural or non-agricultural surface through a network of drains that are underground pipes or channels open to the surface.
AE5 Chemical fertilizers used in agriculture	Quantity of chemical fertilizers used in agriculture	tons of active substance	Industrial products that according to their content can be nitrogen, phosphate, or potassium, and they can also be mixed as complex fertilizers; they are expressed in the active substance.
AE6 Natural fertilizers used in agriculture	The quantity of natural fertilizers used in agriculture	tons of active substance	Includes manure from all species of animals and birds (fresh or fermented) and manure in liquid form; they are expressed in gross weight.
AE7 Pesticides used in agriculture	Amount of pesticides applied in agriculture	kilograms of active substance	Any substance or mixture of substances, including mixtures thereof with ingredients intended for: use in agriculture, forestry, storage, and other activities; for the purpose of preventing, reducing, removing or destroying pests, pathogens, weeds and other forms of animal or plant life, including viruses harmful to plants and domestic animals and insects and rodents carrying diseases infectious to humans; and products for regulating plant growth, defoliation or splitting. They are reported in the active substance.

**Table 3:** Descriptive statistics.

	GDP	ED	ES	AE1	AE2	AE3	AE4	AE5	AE6	AE7
Mean	3.203524	86,540.43	−0.088598	3,062,052	2,922,847	2,138,644	242,868.6	451,465.6	15,090,391	7,753,977
Median	3.770962	85,500.00	1.817528	3,057,047	2,909,177	2,137,828	249,765.0	426,207.0	15,231,715	6,778,183
Maximum	10.42811	113,420.0	8.521318	3,089,065	2,952,174	2,145,656	249,955.0	749,551.0	17,748,826	15,349,466
Minimum	−5.517394	71,140.00	−12.12447	3,045,114	2,901,003	2,131,524	214,196.0	326,123.0	11,748,140	5,242,655
Std. Dev.	4.262231	12,029.72	6.947009	16,314.28	23,287.25	4976.868	13,421.11	112,420.9	1,495,624	2,511,951
Skewness	−0.511692	0.265891	−0.463185	0.343191	0.381434	0.127028	−1.682727	1.349309	−0.366317	2.001159
Kurtosis	2.697116	2.081902	1.777727	1.424972	1.221576	1.639862	3.894702	4.307029	2.634269	6.065172
Jarque–Bera	1.091592	1.078791	2.254109	2.828841	3.588730	1.834749	11.62149	8.616239	0.642573	24.35492
Probability	0.579380	0.583101	0.323986	0.243066	0.166233	0.399567	0.002995	0.013459	0.725216	0.000005

**Table 4:** Unit root tests.

	ADF Test			PP Test		
	Level	First Difference	I(d)	Level	First Difference	I(d)
GDP	−3.3384 **	-	I(0)	−3.3195 **	-	I(0)
ED	−2.1808	−4.1036 ***	I(1)	−2.1808	−7.0995 ***	I(1)
ES	−1.5008	−4.0560 ***	I(1)	−1.5008	−4.0520 ***	I(1)
AE1	−1.5215	−4.3498 ***	I(1)	−1.6195	−4.3453 ***	I(1)
AE2	−0.8793	−4.0857 ***	I(1)	−0.8793	−4.0857 ***	I(1)
AE3	−0.8060	−4.3459 ***	I(1)	−0.5816	−5.0780 ***	I(1)
AE4	−3.4405 **	-	I(0)	−2.7524 *	-	I(0)
AE5	1.5543	−5.2795 ***	I(1)	1.3250	−5.2795 ***	I(1)
AE6	−2.4337	−4.7596 ***	I(1)	−2.4841	−4.6569 **	I(1)
AE7	−5.0093 ***	-	I(0)	−7.5667 ***	-	I(0)

The Schwarz info criteria was used in determining the exogenous lags. The asterisks ***, ** and * imply that the series is stationary at 1%, 5% and 10% levels of significance, respectively.

**Table 5:** Correlation matrix.

Covariance Analysis: Ordinary										
Sample: 1997–2019										
Correlation										
Probability	GDP	ED	ES	AE1	AE2	AE3	AE4	AE5	AE6	AE7
GDP	1.000									
	-									
ED	−0.007	1.000								
	0.9733	-								
ES	0.190	−0.700	1.000							
	0.3831	0.0002	-							
AE1	−0.188	0.796	−0.921	1.000						
	0.3888	0.0000	0.0000	-						
AE2	−0.091	0.740	−0.925	0.978	1.000					
	0.6786	0.0001	0.0000	0.0000	-					
AE3	0.137	−0.863	0.774	−0.853	−0.793	1.000				
	0.5318	0.0000	0.0000	0.0000	0.0000	-				
AE4	0.451	−0.468	0.783	−0.721	−0.645	0.655	1.000			
	0.0304	0.0242	0.0000	0.0001	0.0009	0.0007	-			
AE5	0.055	−0.655	0.535	−0.666	−0.650	0.819	0.415	1.000		
	0.8009	0.0007	0.0084	0.0005	0.0008	0.0000	0.0486	-		
AE6	−0.180	0.329	−0.654	0.613	0.678	−0.347	−0.406	−0.252	1.000	
	0.4107	0.1251	0.0007	0.0019	0.0004	0.1045	0.0543	0.2449	-	
AE7	−0.521	0.584	−0.745	0.723	0.625	−0.658	−0.898	−0.446	0.360	1.000
	0.0107	0.0034	0.0000	0.0001	0.0014	0.0006	0.0000	0.0328	0.0912	-

**Table 6:** Engle–Granger cointegration tests.

	Model 1		Model 2		Model 3	
	Value	Prob. *	Value	Prob. *	Value	Prob. *
Engle–Granger *tau*-statistic	−4.2806	0.0500	−4.7000	0.0347	−5.4352	0.0156
Engle–Granger *z*-statistic	−20.5469	0.0485	−21.7349	0.0393	−25.8365	0.0138

Note: * represents the significance level of the probability.
